# Enhancement of Event-Related Desynchronization in Motor Imagery Based on Transcranial Electrical Stimulation

**DOI:** 10.3389/fnhum.2021.635351

**Published:** 2021-03-18

**Authors:** Jiaxin Xie, Maoqin Peng, Jingqing Lu, Chao Xiao, Xin Zong, Manqing Wang, Dongrui Gao, Yun Qin, Tiejun Liu

**Affiliations:** ^1^MOE Key Lab for Neuroinformation, School of Life Science and Technology, University of Electronic Science and Technology of China, Chengdu, China; ^2^College of Electronic Engineering, Chengdu University of Information Technology, Chengdu, China; ^3^School of Computer Science, Chengdu University of Information Technology, Chengdu, China

**Keywords:** brain-computer interfaces, motor imagery, transcranial alternating current stimulation, transcranial direct current stimulation, event-related desynchronization

## Abstract

Due to the individual differences controlling brain-computer interfaces (BCIs), the applicability and accuracy of BCIs based on motor imagery (MI-BCIs) are limited. To improve the performance of BCIs, this article examined the effect of transcranial electrical stimulation (tES) on brain activity during MI. This article designed an experimental paradigm that combines tES and MI and examined the effects of tES based on the measurements of electroencephalogram (EEG) features in MI processing, including the power spectral density (PSD) and dynamic event-related desynchronization (ERD). Finally, we investigated the effect of tES on the accuracy of MI classification using linear discriminant analysis (LDA). The results showed that the ERD of the μ and β rhythms in the left-hand MI task was enhanced after electrical stimulation with a significant effect in the tDCS group. The average classification accuracy of the transcranial alternating current stimulation (tACS) group and transcranial direct current stimulation (tDCS) group (88.19% and 89.93% respectively) were improved significantly compared to the pre-and pseudo stimulation groups. These findings indicated that tES can improve the performance and applicability of BCI and that tDCS was a potential approach in regulating brain activity and enhancing valid features during noninvasive MI-BCI processing.

## Introduction

Brain-computer interface technology based on motor imagery (MI-BCI) has played an important role in improving and restoring human motor function by activating brain plasticity to induce patients to recover motor control function (Decety and Boisson, 1990). However, studies have shown that individuals differ in their ability to control the BCI. Approximately 15–30% of people could not operate the BCI system effectively, which indicated that their accuracy is lower than that of the majority of people and that they need more training time (Guger et al., 2003). Therefore, it is very important to find methods to improve the applicability of the MI-BCI system and the classification accuracy of electroencephalogram (EEG).

Previous studies have used invasive or noninvasive neural regulation technology to reversibly regulate the activity state of the central nervous system, peripheral nervous system, or autonomic nervous system *via* electrical stimulation or drug regulation to enhance the decoding accuracy and applicability of BCI (He et al., 2015; Cho et al., 2016). Among them, transcranial electrical stimulation (tES; Kuo and Nitsche, 2012; Bestmann and Walsh, 2017), as a non-invasive neuromodulation technique, has attracted considerable attention in recent years. At present, tES mainly adopts transcranial direct current stimulation (tDCS; Unal and Bikson, 2018) and transcranial alternating current stimulation (tACS; Paulus, 2011). According to the polarity of stimulation, an anode is placed on or inside the cortex for tDCS and subthreshold direct current stimulation is introduced to regulate neural activity (Wei et al., 2013; Flöel, 2014). Studies have found that the application of a weak direct current through a scalp electrode could affect the action potential threshold of neurons, increase the activity of spontaneous neurons and then noninvasively regulate the excitability of the cerebral cortex (Bindman et al., 1964; Nitsche and Paulus, 2000; Tsuiki et al., 2019). tACS applies a low-intensity alternating current to the cerebral cortex to regulate the activity of the intracranial central nerve (Kasten and Herrmann, 2017). Ten Hertz tACS stimulation in the primary motor cortex could promote the excitability of the motor cortex, although other frequencies had difficulty evoking excitability changes (Wach et al., 2013).

Studies have shown that tES could effectively regulate brain activities in working memory (Talsma et al., 2017), perception, motor learning, motor control, and other cognitive functions (Nitsche and Paulus, 2000; Angelakis and Liouta, 2011). Therefore, researchers proposed using tES in the BCI system to enhance the excitability of the cerebral cortex and improve the performance of the BCI system (Thomas and Roi, 2018). Baxter et al. (2016) used tDCS in an MI-BCI system and found that although tDCS can improve motor learning ability, cathode stimulation can reduce the power of the α and β bands in the process of right-hand imagery tasks. However, anode tDCS could induce a significant change in the μ rhythm ERD mode, which can conditionally improve the performance of BCI (Wei et al., 2013). Also, several articles have studied the modulation of tACS on motor learning ability (Pollok et al., 2015; Sugata et al., 2018) and showed that the capacity for motor learning was significantly increased for 70 Hz tACS (response time was 270 ms) compared to sham stimulation (response time was 340 ms; Sugata et al., 2018). The application of alpha frequency (7–13 Hz) tACS induced a leftward bias in visuospatial attention relative to the sham (*P* < 0.001; Schuhmann et al., 2019). In addition, applying tACS in the mental rotation task experiment significantly decreased the subject’s alpha and beta rhythm stimulation shortened response time (before_alpha = 0.37 s, before_beta = 0.39 s, after_alpha = 0.3 s, after_beta = 0.34 s; Zhang et al., 2016).

In conclusion, tES could promote motor learning, motor control, MI behavior, and other cognitive functions by regulating the excitability of the cerebral cortex. As a noninvasive stimulation technology, BCIs may be easily accepted. However, previous studies only discussed the effectiveness of a single stimulus mode in BCI systems and did not compare and analyze the stimulus modes that can improve the applicability and effectiveness of BCI systems in the same task. In this article, we designed an experimental paradigm that combines two different modes of stimuli within the same framework and quantified the changes in EEG *via* three measurements from spatial, temporal, and classification dimensions to detect the type of stimulus that can effectively enhance ERD and BCI performance during MI. Here, tDCS and tACS were applied to the Cz position of the subjects’ brains to regulate brain activity and feature extraction was combined with power spectral density (PSD; Liu et al., 2013) and common spatial pattern (CSP; Tariq et al., 2019). Finally, the two features with the largest power difference were extracted by CSP, and the feature vectors were classified by linear discriminant analysis (LDA; Tariq et al., 2020).

## Experiment Preparation

### Subjects

This experiment recruited 15 male college students (23–25 years old, average 24.4 ± 0.44). All the participants were right-handed. None of them had any history of nervous system disease or received any acute or chronic drugs that affected the central nervous system. Written informed consent according to the Declaration of Helsinki was obtained from all participants. This study was approved by the Ethics Committee of the University of Electronic Science and Technology of China (UESTC).

### Signal Acquisition

**Figure 1:**
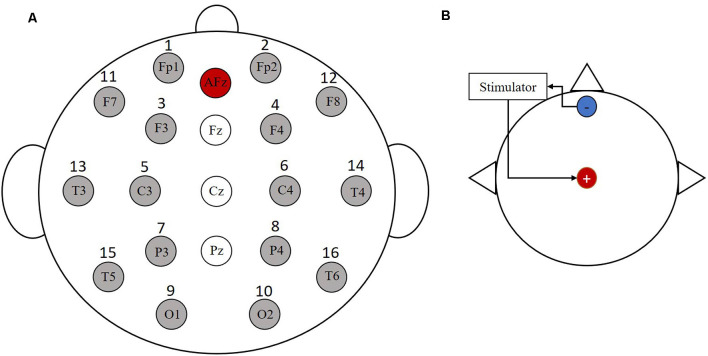
Electrode distribution and stimulation electrode location map. **(A)** The 16 Ag/AgCl electrodes were employed for data recording. **(B)** The anode is placed at the Cz and the cathode is placed at the forehead area.

In this experiment, 16 Ag/AgCl electrodes (i.e., Fp1, Fp2, F3, F4, C3, C4, P3, P4, O1, O2, F7, F8, T3, T4, T5, T6) were employed for data recording using a Symtop amplifier (Symtop Instrument, Beijing, China). The placement of each electrode was determined by the international 10-20 system electrode position method. The electrode distribution diagram is shown in Figure 1A. The reference electrode in this experiment was at the AFz location. Also, the sampling frequency was 1,000 Hz and the impedance was kept below 5 KΩ.

### Electrical Stimulation

Before the subjects performed the MI task, they all randomly underwent three stimulation experiments: tDCS, tACS, and pseudo stimulation, with each stimulation lasting for 10 min. Pseudo stimulation was used as the control to eliminate the placebo effect. The electrode placement position for electrical stimulation is shown in Figure 1B, where the anode was placed at Cz and the cathode was placed at the forehead area. The current intensity of tDCS was 1 mA, the stimulation frequency of tACS was 10 Hz, and the stimulation intensity was determined by the specificity of the subjects (increasing the current intensity gradually in a step size of 0.05 mA from 0 to the intensity at which the subject indicated a stinging sensation or eye pressure flashing). Then, the current intensity at that moment was recorded as the stimulation threshold in the formal experiment (the intensity was not higher than 2 mA).

### Experimental Paradigm

In this article, we designed an experimental paradigm that combines electrical stimulations with MI-BCIs. The MI experiment was conducted in an exclusive room with soft luminance light and a comfortable temperature. One day before the MI experiment, the experimenter asked the subjects to pay attention to certain tasks, including having good rest at night, refusing psychotropic drugs, and maintaining a healthy life. Before the start of the formal experiment, the subjects were sat down in front of the lab computer and explained the procedure of the experiment, and then they signed relevant consent.

**Figure 2:**
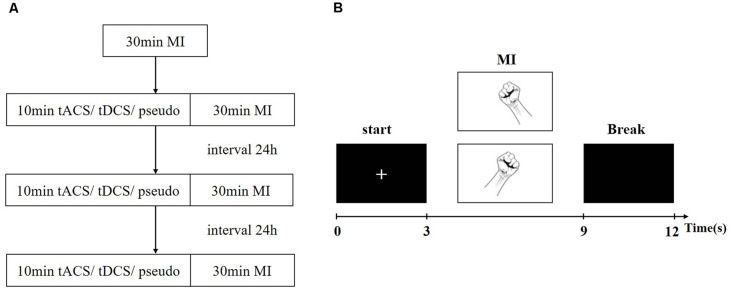
Experiment paradigm. **(A)** The experimental flow chart. **(B)** The Single-trial experiment process.

To familiarize the subjects with the experimental process, they were asked to practice the MI experiment with 40 trials before the formal experiment. During the experiment, participants performed a total of four 30-min MI task experiments and received one kind of stimulation, namely, pseudo stimulation, tDCS, or tACS. The entire experimental flow chart and single-trial design are shown in Figure 2. First, the subjects performed a set of motor imagery EEG experiments before tES, which consisted of 80 trials. The EEG data obtained in this group were used to determine the baseline level of all subjects. Second, the subjects randomly received electrical stimulation lasting for 10 min. During the period of stimulation, there was no EEG acquisition. After stimulation, the subjects were asked to perform another group of MI EEG experiments (all MI EEG experiments’ conditions were the same, including the MI tasks, experimental trials, and duration time). To avoid the post effect of tES, the time interval between each stimulation experiment was at least 24 h.

In one trial, a prompt “+” first appeared on the screen to remind the subjects that the task was about to start. Second, pictures of a left- or right-hand fist appeared on the screen randomly, prompting the subjects to carry out the corresponding left- or right-hand MI, which lasted 6 s. Finally, the screen turned black for 3 s, reminding the subjects to take a break. Each experiment performed a total of 80 trials, including 40 trials for the left-hand task and 40 trials for the right-hand task.

## Materials and Methods

**Figure 3:**
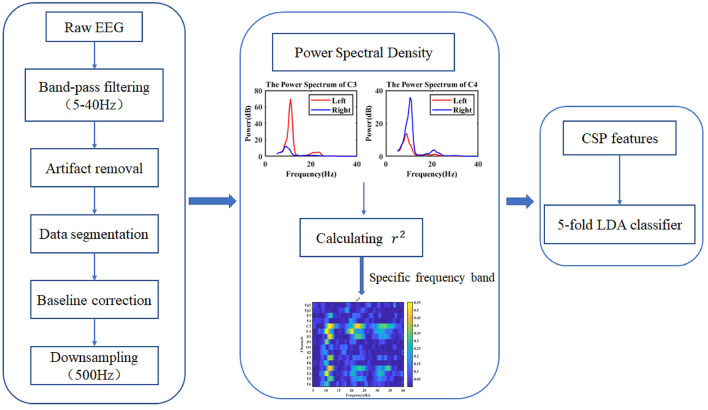
Overall diagram of electroencephalogram (EEG) signal processing.

To study the effect of tES on ERD based on MI, EEG data in four different conditions (prestimulation, pseudo stimulation, tACS, and tDCS) were collected. After EEG preprocessing, the power spectrum of the EEG was calculated and used to extract the specific frequency band, which could represent the greatest difference between the left- and right-hand MI tasks. Then, dynamic ERD based on sliding time windows was obtained. The EEG features in individual EEG frequency bands were extracted using the CSP algorithm and applied in the following pattern recognition classification. The classification accuracy of the left- and right-hand MI of the subjects in each condition was obtained, and the effect of tES on the performance of the MI-BCI was evaluated. The overall implementation steps are shown in Figure 3.

### Signal Preprocessing

Preprocessing aims to obtain effective and reliable EEG trials. The specific steps are as follows: convert the raw data to average reference; filter the data with a 5–40 Hz bandpass filter to obtain the relevant frequency band information; set the threshold to ± 100 μV (according to the EEG amplitude range, the trial with more than 100 μV is considered as a bad trial; Goh et al., 2017); reject data with extreme values; process the remaining data by FastICA to avoid interference by the electrooculogram (EOG) and electromyogram (EMG) artifacts; segment data within the period of [−1, 9 s], in which [−1, 0 s] was considered the baseline for data correction; and downsample the signal to 500 Hz. During the processing of FastICA, the typical characteristics of EOG and EMG were considered. As for EOG (Nguyen et al., 2012), the low frequency-dominated power distribution was always observed in the prefrontal electrodes, while EMG was distributed above 20 Hz, and can be found in most electrodes (Goncharova et al., 2003). After the EOG and EMG components were identified and removed, the pure EEG data was reconstructed.

### Calculation of ΔPower

When imagining the movement of different parts of the body, differences are observed in the spatial distribution of the ERD obtained from the EEG signal. For example, when imagining the movement of the left hand, the ERD phenomenon in the right motor cortex was more significant, in which the electrode with maximum discriminatory power was C4, while when imagining the movement of the right hand, the ERD phenomenon prominent area was in the left-brain area, and the electrode was C3. Therefore, in this article, the μ and β rhythm power were extracted from the C3 and C4 power spectra for each trial of each subject and stimulation condition. First, the PSD of all trials (6 s for each trial) was calculated based on the Pwelch method (Blankertz et al., 2010). For μ rhythm power, the frequency range is 8–13 Hz, and for β rhythm power, the frequency range is 17–25 Hz. Then, we obtained the power difference of C3 and C4 under the same conditions (tasks, rhythms, and stimulation were the same), and the calculation was as follows:

(1) ΔPower=OSP−SSP

where OSP is the PSD of the contralateral electrode during the MI tasks, SSP is the PSD of the ipsilateral electrode, and ΔPower is the difference between the contralateral and ipsilateral sides. For example, to obtain ΔPower during the left-hand MI task, C4 is on the contralateral side and C3 is on the ipsilateral side. Then, normalized ΔPower was obtained:

(2) $$normalized\;\Delta \;Power=\frac{\Delta Power}{OSP+SSP}$$

This step aims to eliminate individual differences.

### Feature Extraction

The CSP method is currently considered the most suitable algorithm for processing the two-category feature extraction of EEG signals (Lu et al., 2010). It is very suitable for processing multidimensional signals and data. By using the spatial correlation of the EEG signal synchronously, the noise of the signal can be eliminated and localization of local cortical nerve activity can be achieved.

**Figure 4:**
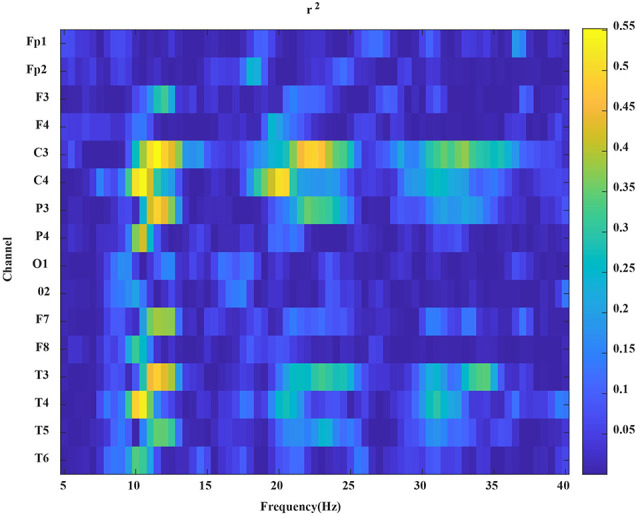
An example of the *r*^2^ map. The *x*-axis represents the frequency, the *y*-axis represents the channels.

From the PSD results, the power spectra of all channels of the subjects in the MI task were estimated. Then, to determine the individual-specific bandpass filter, we calculated the *r*^2^ relative to the two-hand MI tasks for each subject (Xu et al., 2011). *r*^2^ was described as follows:

(3) $$r^{\text{2}}={\left(\frac{\sqrt{N_{1}N_{2}}}{N_{1}+N_{2}}\frac{MEAN\left(P_{1}\right)-MEAN\left(P_{2}\right)}{STD\left(P_{1}UP_{2}\right)}\right)}^{2}$$

where *N*_1_ and *N*_2_ represent the number of trials (both *N*_1_ and *N*_2_ are 40); *P*_1_ and *P*_2_ are the power spectra of EEG data of left and right hand MI tasks, respectively. In the equation above, a larger value of *r*^2^ corresponds to a greater power difference between the EEG data of left- and right-hand MI tasks in this frequency band. In Figure 4, according to the value of *r*^2^, we can select the appropriate bandpass filter frequency band and apply a specific bandpass filter to the MI EEG data.

The CSP algorithm was used to extract features from the processed EEG signals. By designing the parameters of the spatial filter, the best projection matrix *W* was obtained. The EEG signal passed through the spatial filter to obtain the feature vectors that represent the characteristics of left and right signals, one of which has the largest variance and the other has the smallest variance. Finally, the two types of signals were classified by classification algorithms. The specific algorithm processes are as follows (Muller-Gerking et al., 1999):

Note: in the following expressions, *i* represents the MI task category, *i* = 1, 2. It is stipulated that *i* = 1 is left-hand movement, and *i* = 2 is right-hand movement.

Assume that *X*_1_ and *X*_2_ are the single-trial EEG matrices for the left and right hand MI tasks under the same experimental conditions. The matrix dimension is N*T, where *N* is the number of EEG channels, and *T* is the number of sampling points (N ≤ T). *Y*_1_ and *Y*_2_ are two types of MI tasks. In the case of ignoring noise interference, *X*_1_ and *X*_2_ are expressed as follows:

(4) $$X_{i}=\left[A_{i}A_{m}\right]\left[\begin{array}{c} Y_{i}\\ Y_{M} \end{array}\right],$$

where *Y*_M_ is the common source signal of two tasks. The left-hand movement *Y*_1_ and right-hand movement *Y*_2_ source signals of these two tasks are assumed to be linearly independent of each other, and *Y*_1_ and *Y*_2_ are composed of *m*_1_ and *m*_2_, respectively.

The covariance matrix of *X*_1_ and *X*_2_ is calculated as follows:

(5) $$R_{i}=\frac{X_{i}X_{i}^{T}}{tr\left(X_{i}X_{i}^{T}\right)},$$

where *tr* represents the trace of the matrix, which is the sum of the diagonal elements of the matrix *XX*^T^, and *R*_i_ is the covariance matrix of a single trial. According to the total trial *n*_i_, the average covariance matrix $\bar{R}$ is as follows:

(6) $${\bar{R}}_{i}=\frac{1}{n_{i}}\sum_{j=1}^{n_{i}}R_{ij}.$$

The mixed space covariance matrix *R* is as follows:

(7) $$R={\bar{R}}_{\text{1}}+{\bar{R}}_{\text{2}},$$

where *R* is a positive definite matrix, and eigenvalue decomposition is performed on *R* according to the singular value theorem:

(8) $$R=U\lambda U^{T},$$

where λ is a diagonal matrix composed of the eigenvalues arranged in descending order and *U* is the matrix composed of the eigenvectors corresponding to the eigenvalues after decomposition. The whitened matrix *P* is obtained as follows:

(9) $$P=\frac{1}{\sqrt{\lambda }}U^{T},$$

(10) $$Y_{i}=P{\bar{R}}_{i}P^{T}.$$

Then, decomposing the principal component of the whitened matrix obtains the following:

(11) $$Y_{i}=Q_{i}\lambda_{i}Q_{i}^{T},$$

where *Y*_1_, *Y*_2_ have the same eigenvector. The sum of the diagonal matrix of two eigenvalues λ_1_ and λ_2_ is the identity matrix:

(12) $$\lambda_{\text{1}}+\lambda_{\text{2}}=E.$$

The projection matrix *W* can be obtained through the eigenmatrix *Q* and the whitened matrix:

(13) $$W=Q^{T}P,$$

where the projection matrix *W* is the required spatial filter. The EEG matrix *X*_i_ is projected through the spatial filter *W*, and the characteristics can be obtained:

(14) $$Z_{i}=WX_{i}.$$

To avoid the instantaneous change caused by body motion, the variance of the feature signal obtained through the spatial filter is calculated and normalized, and then the feature vector *f*_i_ is extracted as follows:

(15) $$\{\begin{array}{c} Z_{\text{1}}=WX_{i}\\ f_{i}=\frac{\mathrm{var}\left(Z_{i}\right)}{\sum \mathrm{var}\left(Z_{i}\right)} \end{array}.$$

### Quantification of ERD

To compare changes in the C3 and C4 amplitudes elicited by different motor imagery tasks, dynamic ERD was quantified as the relative amplitude (RA) to reveal the power decrease and increase in sliding time windows based on the reference baseline (Jeon et al., 2011); and, we segmented the EEG epochs into 1 s time windows.

The calculation of ERD in each time window was as follows:

(16) $$Act_{\left(j\right)}=\frac{1}{N}\sum_{i=1}^{N}y_{ij}^{\text{2}},$$

where, *y*_ij_ is the jth sample of the ith trial, *N* is the number of trials and *Act*_(j)_ is the average power at jth sample squared.

(17) $$R=\frac{1}{k+1}\sum_{j\;=\;r_{\text{0}}}^{r_{\text{0}}+k}Act_{\left(j\right)},$$

where *R* is the average power in the reference interval [*r*_0_, *r*_0_ + *k*]. Due to the great individual difference, in this study, the reference interval adopted the whole time course during MI tasks, i.e., [−1 9 s].

(18) $$RA_{\left(j\right)}\left(\%\right)=\left(\frac{Act_{\left(j\right)}-R}{R}\right)\times \text{100}\%.$$

### Pattern Recognition Classification

Suppose that the dataset D = {(*X*_1_, *Y*_1_), (*X*_2_, *Y*_2_), …, (*X*_m_, *Y*_m_)}, where *X*_i_ is an n-dimensional vector, *y*_i_ ∈ {0, 1}. Here, *N*_j_(*j* = 0, 1) is the number of samples of type *j*, *X*_j_(*j* = 0, 1) is the set, *μ_j_*(*j* = 0, 1) is the mean value, and Σ_j_(*j* = 0, 1) is the covariance matrix.

(19) $$\mu_{j}=\frac{1}{N_{j}}\sum_{x\in X_{j}}x,....j=\text{0},\text{1}.$$

The expression of *μ_j_* is as follows:

(20) $$\Sigma_{j}=\sum_{x\in X_{j}}\left(x-\mu_{j}\right){\left(x-\mu_{j}\right)}^{T},....j=\text{0},\text{1}.$$

The expression of Σ_*j*_ is as follows:

Since there are two types of data, we only need to project the data onto a straight line. Assuming that the projection line is a vector *w*, the projection of any sample on *w* is *w*^T^*x*_i_; the center points μ_0_ and μ_1_ of the two categories, projected on *w* are *w^T^*μ_0_ and *w^T^*μ_1_, respectively. The main purpose of LDA is to maximize the distance between the centers of different categories of data to maximize ${\left\|w^{T}\mu_{\text{0}}-w^{T}\mu_{\text{1}}\right\|}_{\text{2}}^{\text{2}}$. At the same time, we need to make the projection points of the same kind of data as small as possible; that is, the covariance *w^T^Σ_0_w* and *w^T^Σ_1_w* of projection points between similar samples should be as small as possible. Therefore, we need to minimize *w^T^Σ_0_w* + *w^T^Σ_1_w*.

The optimization objective of the LDA algorithm is as follows:

(21) $$W^{*}=\arg\max\frac{{\left\|w^{T}\mu_{\text{0}}-w^{T}\mu_{\text{1}}\right\|}_{2}^{2}}{w^{T}\sum_{\text{0}}w+w^{T}\sum_{\text{1}}w}=\frac{w^{T}\left(\mu_{\text{0}}-\mu_{\text{1}}\right){\left(\mu_{\text{0}}-\mu_{\text{1}}\right)}^{T}w}{w^{T}\left(\sum_{\text{0}}+\sum_{\text{1}}\right)w}.$$

The between-class scatter matrix *S*_B_ is as follows:

(22) $$S_{B}=\left(\mu_{\text{0}}-\mu_{\text{1}}\right){\left(\mu_{\text{1}}-\mu_{\text{0}}\right)}^{T}.$$

The within-class scatter matrix *S*_W_ is as follows:

(23) $$S_{W}=\sum_{\text{0}}+\Sigma_{\text{1}},$$

Therefore, the W* is rewritten as follows:

(24) $$W^{*}=\arg\max\frac{w^{T}S_{B}w}{w^{T}S_{W}w}.$$

Since both the numerator and denominator contain the quadratic term of *w*, the objective function is independent of the module length of *w*. Let:

(25) $$w^{T}S_{W}w=\text{1}.$$

The optimization problem is as follows:

(26) $$\{\begin{array}{c} \min_{\left(W\right)}-w^{T}S_{B}w\\ s.t.\;w^{T}S_{W}w=\text{1} \end{array}.$$

The Lagrangian function of the optimization problem is as follows:

(27) $$L\left(w,\lambda \right)=-w^{T}S_{B}w+\lambda \left(w^{T}S_{W}w-\text{1}\right),$$

Then, by finding the first partial derivative of *w* for equation (20) and setting it to zero, we can obtain the following:

(28) $$S_{W}^{-\text{1}}S_{B}w=\lambda_{w}.$$

By finding the eigenvector of the matrix, we can obtain *w*.

### Statistical Analysis

Group-level statistical tests were conducted for different EEG measurements, including the normalized ΔPower, dynamic ERD, and classification accuracy. Before the statistical tests, the data distribution was first examined based on Mauchly’s test of sphericity. Then, a repeated measurement variance analysis of the general linear model was performed for each group to test the significance among the subjects in different experimental conditions. For the normalized ΔPower, one-way repeated-measures analysis of variance (ANOVA) and *post hoc* *t*-tests were performed on the power for the μ and β rhythms of the left- and right-hand Ml tasks in four conditions. To obtain the optimal time range, the values of ERD difference between the contralateral and ipsilateral sides of all the time windows were compared between pre-and poststimulation in left- and right-hand sides (paired *t*-test). For the classification accuracy-the significance of differences among experimental conditions was also tested *via* ANOVA. All statistic thresholds were set to *P* < 0.05 without correction.

## Results and Analysis

### Analysis of the Power Spectrum Characteristics

**Figure 5:**
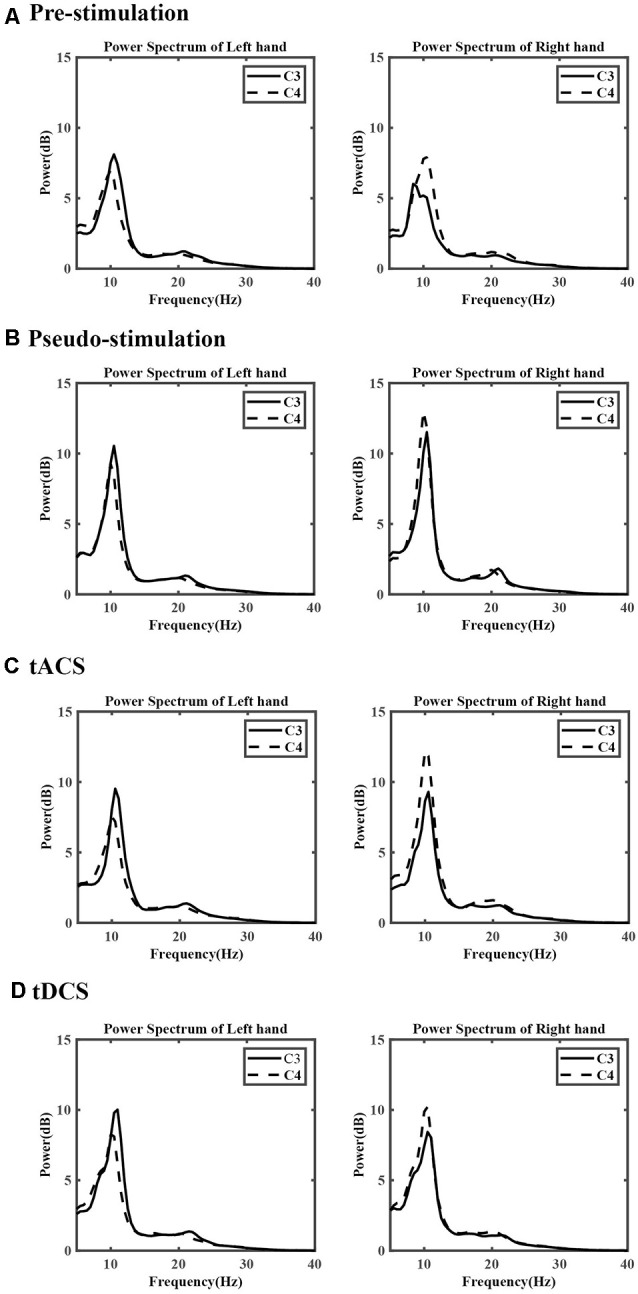
The average power spectrum of each group. **(A)** The prestimulation group, **(B)** the pseudo stimulation group, **(C)** the transcranial alternating current stimulation (tACS) group, and **(D)** the tDCS group. The *x*-axis represents the frequency, the *y*-axis represents the power.

The power change of the μ and β rhythms among the sensory-motor rhythms (SMRs) during the left- and right-hand MI tasks was calculated according to the average power spectrum collected by the C3 and C4 channels for all subjects in the four experimental conditions. The results are shown in Figure 5. For the power change of the μ rhythm at 8–13 Hz, during the right-hand MI tasks, the power of C3 was lower than that of C4 both in pre-and poststimulation. When subjects performed left-hand MI tasks, obvious power differences were not observed between C3 and C4 in the prestimulation and pseudo stimulation groups. However, after tACS and tDCS, the phenomenon could be observed obviously. For the β rhythm of 17–25 Hz, the power change in the C3 and C4 regions was slight.

**Figure 6:**
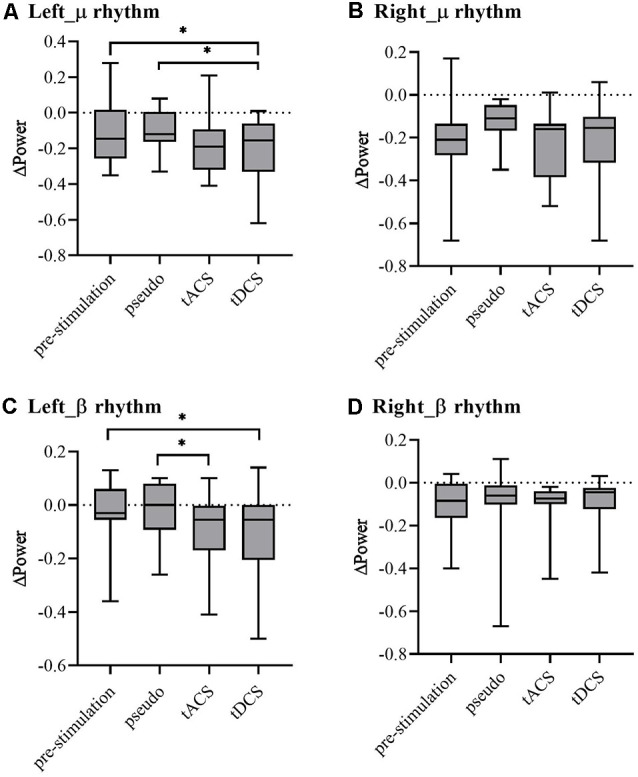
The ΔPower spectrum of each group (Note: ΔPower is the difference of ipsilateral and contralateral sides). **(A)** μ rhythm of the left-hand task, **(B)** μ rhythm of the right-hand task, **(C)** β rhythm of the left-hand task, and **(D)** β rhythm of the right-hand task. * represents significance *P* < 0.05.

To compare the power change statistically, in Figure 6, the μ and β rhythm power were extracted from the C3 and C4 power spectra for each trial of each subject and stimulation condition. ANOVAs were performed on the ΔPower for μ and β rhythm power of left- and right-hand MI tasks in four experiments to evaluate the reference effects. Significant differences revealed by ANOVA were further analyzed for multiple comparisons using Tukey’s *post hoc* test. For left-hand tasks, the μ and β rhythms were in line with the Mauchly sphere test hypothesis (*P* = 0.05). For the μ rhythm, compared with the pre- and pseudo stimulation groups, the tDCS group showed significant differences but the tACS group showed almost no significant change (tDCS-pre: *P* = 0.03 < 0.05; tDCS-Pseudo: *P* = 0.01 < 0.05). For the β rhythm, tDCS showed significant differences compared with prestimulation and tACS showed significant differences compared with pseudo stimulation, while tDCS showed marginal significance (tDCS-Pre: *P* = 0.01 < 0.05; tACS-Pseudo: *P* = 0.01 < 0.05; tDCS-Pseudo: *P* = 0.05). For right-hand tasks, the μ and β rhythms were in line with the Mauchly sphere test hypothesis (*P* = 0.05). Neither tDCS and tACS were significantly different.

### Analysis of Event-Related Desynchronization Features

**Figure 7:**
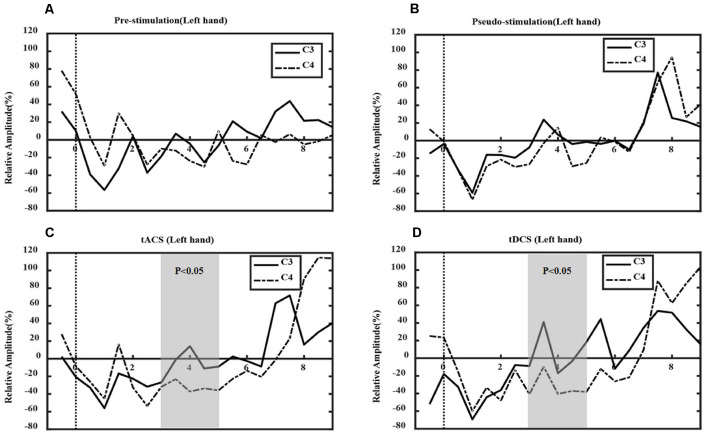
Event-related desynchronization (ERD) in left-hand tasks. Where the *x*-axis represents time and the *y*-axis represents relative amplitude (RA). **(A)** The prestimulation group, **(B)** the pseudo stimulation group, **(C)** the tACS group, and **(D)** the tDCS group. The RA in tACS and tDCS groups are smaller than in prestimulation and pseudo stimulation groups. The optimal time window is [3, 5 s] (tACS: *P* = 0.03 < 0.05; tDCS: *P* = 0.03 < 0.05).

**Figure 8:**
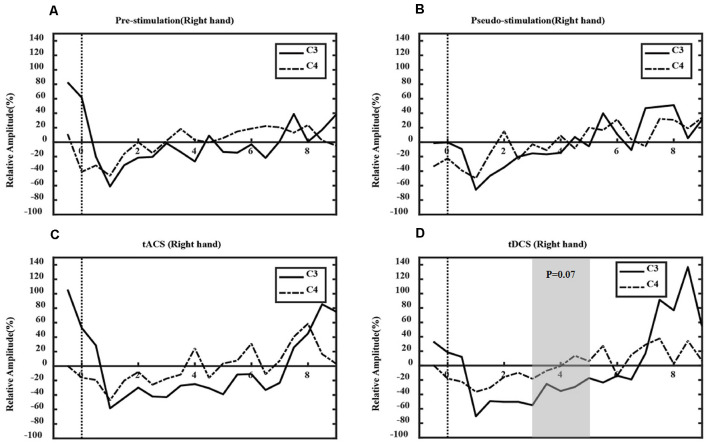
ERD in right-hand tasks. Where the *x*-axis represents time and the *y*-axis represents RA. **(A)** The prestimulation group, **(B)** the pseudo stimulation group, **(C)** the tACS group, and **(D)** the tDCS group. The RA in tACS and tDCS groups are smaller than in pre-stimulation and pseudo-stimulation groups. The optimal time range is [3, 5 s] (tDCS: *P* = 0.07 > 0.05).

Figures 7, 8 show the ERD relative amplitude (RA) time courses of all subjects from [−1, 9 s] [from 1 s before the MI task (6 s) to 3 s after the task] for the two MI tasks. A smaller RA of the contralateral side corresponded to greater desynchronized ERD movement. In this study, different ERD phenomena were observed. In Figure 7, the duration of C4 ERD in the tACS and tDCS groups was longer than that in the pre-and pseudo stimulation groups. Also, the RA in the tACS and tDCS groups was smaller than that in pre-and pseudo stimulation groups. In Figure 8, the duration of C3 ERD in the tACS and tDCS groups was longer than that in pre- and pseudo stimulation groups. The RA in the tACS and tDCS groups was smaller than that in pre- and pseudo stimulation groups.

Also, the optimal time range of ERD was verified. For the left-hand tasks, we used one-way ANOVA to test the significance of the RA difference between the contralateral and ipsilateral sides in different experimental conditions, and the optimal time window [3, 5 s] was found (tACS-Pre: *P* = 0.03 < 0.05; tDCS-Pre: *P* = 0.03 < 0.05). However, in the right-hand tasks, the optimal time range was [3–5 s], with statistical significance (tACS-Pre: *P* = 0.13 > 0.05; tDCS-Pre: *P* = 0.07 > 0.05). Compared with prestimulation, tDCS presented marginal significance; however, tACS was not significant.

### Analysis of Classification Accuracy

**Figure 9:**
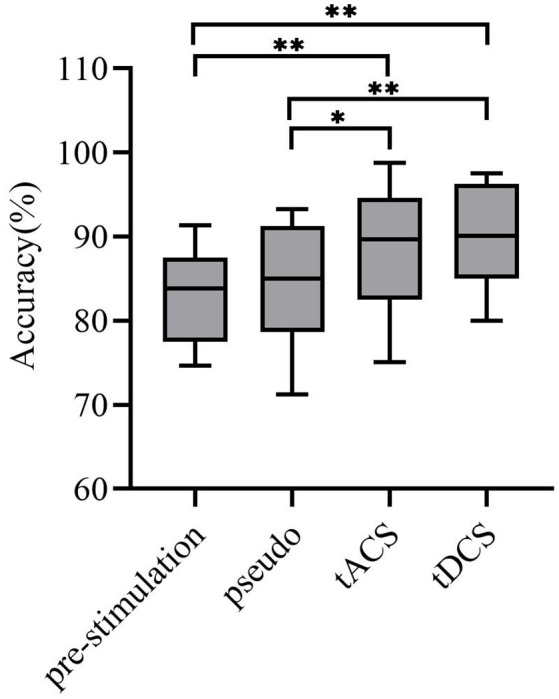
Average classification accuracy. Where the *x*-axis represents experiment modes and the *y*-axis represents accuracy. The average classification accuracy of the tACS group and tDCS group was improved significantly (tACS-Pre: **P < 0.001; tDCS-Pre: **P < 0.001; tACS-Pseudo: *P < 0.05; tDCS-Pseudo: **P < 0.001). * and ** represent significance.

In this article, the LDA classifier was used to train and test the MI classification of subjects under four different conditions: prestimulation, pseudo stimulation, tACS, and tDCS. After preprocessing the data, we determined the individual specific bandpass filter of the two tasks, and the results showed that nine subjective specific frequency bands were from 8–15 Hz, four subjective specific frequency bands were from 17–25 Hz, and two subjective specific frequency bands were from 8–30 Hz. Then, the filtered data were extracted by CSP and the two types of extracted feature vectors *f*_1_ and *f*_2_ were input into the LDA classifier as training data for classification. According to the relatively small distance between similar data points and the relatively large distance between data points of different classifications, the best separation plane was obtained. Then the 5-fold cross-validation method was implemented for training and testing. The final classification accuracy results of all subjects are shown in Table 1. The average classification accuracy of the four groups of experiments is shown in Figure 9.

**Table 1:** The classification accuracy of subjects’ motor imagery.

Subjects	Pre-stimulation (%)	Pseudo-stimulation (%)	tACS (%)	tDCS (%)
Subject 1	91.25	93.21	98.75	96.88
Subject 2	83.83	85.84	92.50	97.50
Subject 3	74.68	76.25	86.25	80.00
Subject 4	85.00	85.00	89.74	90.06
Subject 5	82.49	85.00	80.00	86.25
Subject 6	77.50	78.64	85.05	82.68
Subject 7	77.61	71.25	81.27	87.49
Subject 8	77.49	77.50	75.11	81.27
Subject 9	88.77	91.28	94.93	96.31
Subject 10	87.5	92.5	94.64	97.5
Subject 11	91.32	92.78	89.64	92.7
Subject 12	84.29	80.2	95	95
Subject 13	83.55	87.34	87.5	90
Subject 14	74.78	82.06	82.5	85
Subject 15	85.12	87.98	90	90.28
Mean ± Standard	83.01 ± 1.43	84.46 ± 1.73	88.19 ± 1.70	89.93 ± 1.56

From the data in the table, the average accuracy of MI was effectively improved after the subjects received tDCS. For tACS, the accuracy of subjects 5, 8, and 11 after tACS decreased compared with that before stimulation. Subjects 1, 3, and 6 had better accuracy improvement with tACS than those with tDCS. Among all subjects in the experimental group, the highest accuracy of 98.75% was found for subject 1 after tACS, and the lowest accuracy of 75.11% was observed in subjects 8 after tACS. Figure 9 shows that the overall classification accuracy of the tACS group and the tDCS group was significantly improved compared to that of the pre-and pseudo stimulation groups, although the improvement effect of the tDCS group was higher than that of the tACS group.

To investigate the effect of the tACS and tDCS proposed in this article for improving the accuracy of MI classification tasks, we used one-way ANOVA to test the significance of the MI classification accuracy of the subjects under different experimental conditions (*P* = 0.05). First, we confirmed the homogeneity of the sample’s variance, which is consistent with the Mauchly sphere test hypothesis (*P* = 0.134 > 0.05), thus demonstrating that the main effect is significant. Second, the results of tACS and tDCS were compared with the results of the pre-and pseudo stimulation groups, respectively. These statistics revealed significant differences for all accuracies compared with the pre-and pseudo stimulation groups (tACS-Pre: *P* < 0.001; tDCS-Pre: *P* < 0.001; tACS-Pseudo: *P* < 0.05; tDCS-Pseudo: *P* < 0.05). Therefore, we concluded that the accuracy of the subjects in the tACS and tDCS groups was significantly improved compared with that of the pre-stimulation and pseudo stimulation groups.

## Discussion

The main purpose of this article was to study the effect of tES on ERD based on MI-BCI. Subjects performed the MI classification experiment under four conditions: prestimulation, pseudo stimulation, tDCS, and tACS. The effects of tACS and tDCS on ERD were analyzed from three aspects: power spectral density, dynamic ERD, and classification accuracy. Also, the average classification accuracy was used to verify the improvement of BCI task ability.

Motor imagery was described as imagining a movement rather than executing a real movement, and this method is promising for patients with tetraplegia, spinal cord injury, and amyotrophic lateral sclerosis (ALS; Abiri et al., 2019). However, the main drawback of the MI was that the training time could take weeks or months. tACS and tDCS as noninvasive neuromodulation techniques could provide alternative ways to enhance the valid metrics by modifying ERD patterns (Kuo and Nitsche, 2012). Whether for motor execution or MI, ERD changes in SMRs are always produced (Jeon et al., 2011; Bauer et al., 2015). Among them, the μ rhythm and β rhythm among SMRs were considered to be related to motor ability and motor control (Pfurtscheller et al., 1994). In the process of the unilateral MI task, the power of the μ rhythm and β rhythm decreased in the contralateral motor-sensory area, namely, the ERD (Pfurtscheller and Neuper, 1994, 1997). Many articles have indicated that tES could modulate ERD during MI. In this study, some interesting findings were obtained: (1) tES could induce both μ rhythm and β rhythm ERD increases in the left-hand MI task; (2) tES can prolong the ERD duration and decrease the relative power; and (3) tES can enhance the MI accuracy effectively.

These findings could provide a reference for related fields. Studies have indicated that there is differential lateralization of hand movement neural representation in right- and left-handed individuals, and handedness is closely linked to the ability to control an SMR-BCI (Zapała et al., 2020). In the current study, all the subjects recruited were right-handed, power suppression of the μ rhythm occurred during right-hand MI of all conditions, but there was no significance between pre-and poststimulation. However, in the left-hand MI task, the power of the μ rhythm declined obviously after tDCS, and the ΔPower of the μ rhythm decreased significantly compared with pre-and pseudo stimulation (tDCS-pre: *P* = 0.03 < 0.05; tDCS-Pseudo: *P* = 0.01 < 0.05). These results implied that tES may evoke a much higher effect on the neural representation of the non-dominant hand MI task. Also, as one important motor rhythm, the power of the β rhythm was not significant in this study. Interestingly, the ΔPower of the β rhythm in the left-hand MI showed a significant decrease after tDCS compared with pre-stimulation (*P* < 0.05), indicating that this power difference between bilateral electrodes may create the potential control signal that drives a BCI. Moreover, the relative amplitude of ERD during MI was enhanced after tACS and tDCS, indicating that transcranial electrical stimulation can enhance the excitability of the cerebral cortex and regulate brain activity (Pellicciari et al., 2013). Although the duration of ERD in tACS and tDCS seemed to have been prolonged, not all the whole period of MI in each trial was usable. The effective response time of MI was different among individuals, due to the pattern of neural activation (Williams et al., 2012), and a previous study indicated that the overall optimal time segment was [4, 6 s] (Gong et al., 2013). In this article, the optimal time range was [3, 5 s], where the left-hand relative amplitudes of ERD in tACS and tDCS were significant (*P* < 0.05), but the right-hand relative amplitudes of ERD, which were was also the range of [3, 5 s], were not significant after tACS.

To verify the influence of tES on MI-BCI task ability, we compared the classification accuracy of the four conditions. tACS enhanced motor imagery ability in terms of the μ and β rhythm. A possible mechanism was that beta rhythm stimulation was related to the excitability of the primary motor cortex and the alpha rhythm stimulation was associated with motor educability (Zhang et al., 2016). tDCS could enhance ERD patterns and conditionally improve BCI performance in both the online and offline BCI classification results(Wei et al., 2013). In this study, we used CSPs to extract the signal features and LDA to classify the feature vectors (Wang et al., 2005; Sharma and Paliwal, 2015). The results showed that the average classification accuracy of the tACS group and tDCS group was improved significantly (*P* < 0.001). However, individual differences impacted tACS, possibly because of the difference of endogenous oscillations among individuals with tACS frequencies.

In this study, we designed an experimental paradigm that combines two different modes of stimuli and compared them with the stimuli to determine the most effective at enhancing event-related desynchronization during the MI period. In the same processing framework, the comparison analysis of the quantified EEG metrics was conducted from three dimensions including the PSD difference between contralateral and ipsilateral electrodes (spatial effect), the time-varying ERD calculation using sliding windows (temporal effect), and the classification accuracy based on the classical LDA method in MI-BCI (classification performance). From the results of EEG metrics and classification accuracy, we speculated that tDCS has potential in regulating brain activity and enhancing valid features in noninvasive MI-BCI processing. Moreover, the time range of [3, 5 s] after MI start-up led to the optimal ERD combined with tDCS, which may be helpful for the actual BCI performance improvement. However, this study also has many limitations in the experimental and analytical methods. For example, for the experimental design, the duration of electrical stimulation was 10 min and the anode of the stimulation position was located in Cz. In subsequent experiments, different experimental groups could set the stimulation duration to 5, 15, and 20 min, and implement different placement of the anode of tES (Kasashima et al., 2012; Mordillo-Mateos et al., 2012; Wei et al., 2013; Koo et al., 2016). Additionally, the effect of tACS may change due to differences in the endogenous oscillation among individuals. Even if the deviation from the internal frequency of individuals is very small, it may cause other effects or reduce the modulation effect of tACS (Herrmann et al., 2013). The preliminary conclusion based on the findings was that tES may make subjects start MI tasks faster; however, this point requires further investigation. Moreover, the number of subjects should be increased in subsequent experiments to verify the results of the statistical test.

## Conclusion

In this article, tDCS and tACS were conducted and evaluated based on the same motor imagery (MI) tasks and subjects. The two tES methods can effectively enhance the activation of the cerebral motor cortex, which makes ERD more obvious during the MI period. Then, we quantified ERD by dynamic time windows, which can provide the optimal time range of [3, 5 s] for future MI-BCIs. Moreover, in the case of using the basic feature extraction and classification algorithm for EEG signal processing, both kinds of stimulation methods can improve the performance of MI-BCI using a lower difficulty algorithm and tDCS showed superiority in regulating activity and evoking effective features in MI-BCI.

## Data Availability Statement

The raw data supporting the conclusions of this article will be made available by the authors, without undue reservation.

## Ethics Statement

The studies involving human participants were reviewed and approved by Ethics Committee of the University of Electronic Science and Technology of China. The patients/participants provided their written informed consent to participate in this study.

## Author Contributions

JX, MP, DG, and CX conceived and designed the work. JL, XZ, MW, and DG acquired the data. MP, XZ, MW, and CX analyzed the data. JX, MP, MW, and JL wrote the article. All authors revised the work for important intellectual content. All authors contributed to the article and approved the submitted version.

## Conflict of Interest

The authors declare that the research was conducted in the absence of any commercial or financial relationships that could be construed as a potential conflict of interest.
