# Supplementary material for: Enhancement of Event-Related Desynchronization in Motor Imagery Based on Transcranial Electrical Stimulation
Source: Front Hum Neurosci. 2021 Mar 18;15:635351. doi: 10.3389/fnhum.2021.635351 (PMC8012503; doi:10.3389/fnhum.2021.635351)
Supplement: Supplementary file 1 [file Table_1.DOCX]

Supplementary materials

The power of the C3 and C4 channels significantly change pre- and after-stimulation. In order to examine the ERD in C3 and C4 during the MI task of each experiment condition, and time-frequecy analysis was conducted using the EEGLAB toolbox in MATLAB software. The corresponding event-related spectral perturbations (ERSP) were plotted. In this supplementary materials, we have choose subject 1, 5, 8, 10 as the examples, and their time-frequency distributions were shown in Figure 1-8. The ERD phenomenon was found all around 10Hz and 20Hz for all MI tasks, corresponding to the SMR between μ rhythm and β rhythm.

For the left-hand MI task, C3 and C4 were ipsilateral and contralateral electrode respectively. As shown in Figure 1-4, the power in contralateral electrode was significantly decrease after tACS and tDCS.

(A) Pre-stimulation

(B) Pseudo-stimulation

(C) tACS

(D) tDCS

Figure 1. ERSP of subject 1 in left-hand task

(A) Pre-stimulation

(B) Pseudo-stimulation

(C) tACS

(D) tDCS

Figure 2. ERSP of subject 5 in left-hand task

(A) Pre-stimulation

(B) Pseudo-stimulation

(C) tACS

(D) tDCS

Figure 3. ERSP of subject 8 in left-hand task

(A) Pre-stimulation

(B) Pseudo-stimulation

(C) tACS

(D) tDCS

Figure 4. ERSP of subject 10 in left-hand task

For the right-hand MI task, C3 and C4 were contralateral and ipsilateral electrode respectively. As shown in Figure 5-8, the power in C3 electrode was significantly decrease after tACS and tDCS.

(A) Pre-stimulation

(B) Pseudo-stimulation

(C) tACS

(D) tDCS

Figure 5. ERSP of subject 1 in right-hand task

(A) Pre-stimulation

(B) Pseudo-stimulation

(C) tACS

(D) tDCS

Figure 6. ERSP of subject 5 in right-hand task

(A) Pre-stimulation

(B) Pseudo-stimulation

(C) tACS

(D) tDCS

Figure 7. ERSP of subject 8 in right-hand task

(A) Pre-stimulation

(B) Pseudo-stimulation

(C) tACS

(D) tDCS

Figure 8. ERSP of subject 10 in right-hand task
